# Hypermethylated *APC* in serous carcinoma based on a meta-analysis of ovarian cancer

**DOI:** 10.1186/s13048-016-0271-6

**Published:** 2016-09-26

**Authors:** Chunyan Shen, Qifang Sheng, Xiaojie Zhang, Yuling Fu, Kemiao Zhu

**Affiliations:** Obstetrics and Gynecology Department, The No. 2 Hospital of Yinzhou, Ningbo, 315040 Zhejiang China

**Keywords:** *APC*, Hypermethylation, Ovarian cancer, Serous carcinoma, Biomarker

## Abstract

**Background:**

The reduced expression of the Adenomatous polyposis coli (*APC*) gene, a tumor suppressor gene, through promoter hypermethylation has been reported to play a key role in the carcinogenesis. However, the correlation between *APC* promoter hypermethylation and ovarian cancer (OC) remains to be clarified.

**Methods:**

A comprehensive literature search was carried out in related research databases. The overall odds ratio (OR) and corresponding 95 % confidence interval (CI) were used to evaluate the effects of *APC* promoter hypermethylation on OC and clinicopathological characteristics.

**Results:**

Ultimately, 12 eligible studies were used in our study, including 806 OC samples, 429 normal controls, 109 benign lesions and 75 LMP samples. The pooled OR showed that *APC* promoter hypermethylation was significantly higher in OC than in normal and benign controls (OR = 6.18 and OR = 3.26, respectively). No significant correlation was observed between OC and low malignant potential (LMP) tumors (*P* = 0.436). In the comparison of OC and normal controls, subgroup analysis based on race showed that the overall OR of *APC* promoter hypermethylation was significant and similar in Asians and Caucasians (OR = 8.34 and OR = 5.39, respectively). A subgroup analysis based on sample type found that the pooled OR was significantly higher in blood than in tissue (OR = 18.71 and OR = 5.74, respectively). A significant association was not observed between *APC* promoter hypermethylation and tumor grade or tumor stage. The pooled OR indicated that *APC* promoter hypermethylation was significantly lower in serous carcinoma than in non-serous carcinoma (OR = 0.56, *P* = 0.02). No obvious publication bias was detected by Egger’s test (all *P* > 0.05).

**Conclusions:**

*APC* promoter hypermethylation may be linked to the increased risk of OC. It was associated with histological type, but not with tumor grade or tumor stage. Moreover, hypermethylated *APC* may be a noninvasive biomarker using blood samples. Future studies are required to validate these results.

## Background

Ovarian cancer (OC) is the second most common gynecologic cancer after cervical cancer and the most lethal gynecologic cancer [1]. Based on cancer statistics, approximately 22,280 new cases are estimated in 2016 in the USA, leading to 14,240 deaths due to OC [1]. Serous carcinoma is the most common histotype of ovarian cancer and accounts for the majority of deaths [2]. This is possibly due to the lack of symptoms of early-stage disease and effective early detection methods; less than 20 % of ovarian cancer patients can be diagnosed early [3]. The diagnosis of ovarian cancer is frequently determined at an advanced stage and most patients are treated via surgery combined with chemotherapy drugs [4]. The 5-year relative survival rate of ovarian cancer patients is only 38 % [1].

Studies have proven that epigenetic modifications, including DNA methylation, histone modifications, nucleosome positioning and non-coding RNAs, are early and frequent events in cancer [5, 6]. DNA methylation as a major mechanism of epigenetic modifications plays an important role in carcinogenesis and cancer progression [7, 8]. Cancer is a genetic disease that involves abnormalities of oncogenes and/or tumor suppressor genes (TSGs) [9]. Aberrant DNA methylation of CpG islands in the promoter regions leads to the silencing of tumor suppressor genes in cancer [10, 11]. The adenomatous polyposis coli (*APC*) gene is a tumor suppressor gene located on chromosome 5q21 that encodes a large multidomain protein [12]. The dysfunction of the adenomatous polyposis coli (*APC*) protein participates in tumorigenesis [13]. The *APC* gene has a major role in WNT signaling, cell cycle regulation, cell differentiation and proliferation, transcriptional activation, chromosomal instability, and apoptosis [14–16].

However, an individual study with a small number of subjects may lack strong statistical power. Thus, we systematically investigated studies of *APC* promoter hypermethylation and OC to evaluate the correlation between *APC* promoter hypermethylation and OC. Moreover, we also validated the clinicopathological significance of hypermethylated *APC* in ovarian cancer.

## Methods

### Literature search

The relevant literature were found in the PubMed, Embase, EBSCO, Cochrane Library, CNKI and Wanfang databases without language limitations. The following keywords or search terms were used: (adenomatous polyposis coli OR APC) AND (ovarian OR ovary) AND (cancer OR carcinoma OR tumor OR neoplasm) AND (methylation OR epigene*), updated to March 21^st^, 2016.

### Selection criteria

The eligible studies had to meet the following inclusion criteria: 1) cancer patients were diagnosed as primary ovarian carcinoma by histopathological examination; 2) studies were associated with *APC* gene promoter methylation and ovarian cancer; 3) the methylated *APC* gene must have sufficient data about the frequencies of promoter methylation to evaluate the correlation of *APC* promoter methylation and ovarian cancer with clinicopathological features; 4) if the authors used duplicate sample data and published more than one paper, only the most recent paper or the most complete paper with the larger sample size was applied. The excluded studies did not meet the above inclusion criteria.

### Data extraction

For the eligible studies included in the current meta-analysis, the relevant information were extracted as follows: the first author’s surname, publication year, country, ethnic population, methylation detection method, sample type, the number of *APC* promoter methylation events, sample size, and clinicopathological characteristics, such as tumor grade, tumor stage and tumor histology. Benign lesions, normal samples and low malignant potential (LMP) tumors were defined as controls. Moreover, tumor grades of ≤ 2 were defined as low-grade, and a tumor grade of 3 was defined as high-grade. Tumor stages of ≤ 2 were defined as early stage, while tumor stages of 3–4 were defined as advanced stage. Non-serous histotypes consisted of clear cell carcinoma (CC), endometrioid carcinoma (EC), mucinous carcinoma (MC) or transitional cell carcinoma (TC).

### Statistical analysis

The present study was performed using STATA software (version 12.0, Stata Corporation, College Station, TX, USA). The pooled odds ratio (OR) and corresponding 95 % confidence interval (95 % CI) were calculated and summarized to assess the relationship between *APC* promoter methylation and ovarian cancer. Heterogeneity of eligible studies was evaluated based on Cochran’s Q test and I^2^ statistic [17]. If I^2^ ≥ 50 % and *p* < 0.1, significant heterogeneity was observed and a random-effects model was used; otherwise, a fixed-effects model was applied, indicating a lack of heterogeneity [18, 19]. Publication bias was detected using Egger’s linear regression test [20].

## Results

### The characteristics of included studies

**Fig. 1:**
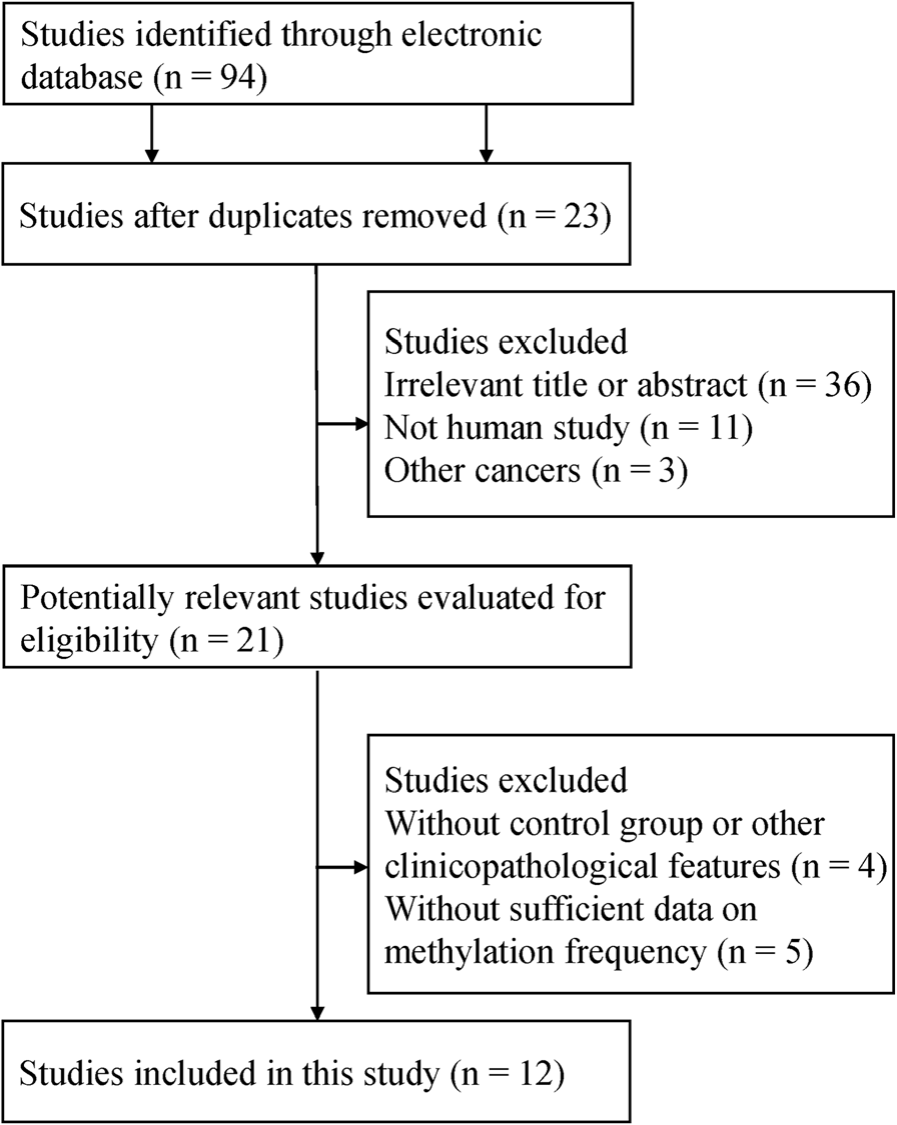
Flow diagram of the literature search strategy to identify studies

Initially, we searched 94 potentially relevant articles using the above databases and keywords. According to the inclusion criteria, the final 12 studies that met the selection criteria were included in the present meta-analysis (Fig. 1). The methylation region was the promoter. Among the 12 studies, only 1 study used quantitative methylation specific PCR (QMSP) detection; the others used the methylation specific PCR (MSP) test. Twelve studies assessed the correlation between *APC* promoter methylation and ovarian cancer, including 10 cancer-normal studies, 7 cancer-benign studies and 5 cancer-LMP studies. In addition, 8 studies evaluated the association between *APC* promoter methylation and clinicopathological features, including 4 studies of tumor grade, 5 studies of tumor stage and 7 studies of tumor histology. The basic characteristics of selected studies were presented in Table 1 [21–32].

**Table 1:** The basic characteristics of the included studies

First author	Country	Ethnicity	Method	Sample	Cancer	LMP	Benign	Normal	Low-grade	High-grade	StageI-II	Stage III-IV	Serous carcinoma	Non-serous carcinoma
					N (M %)	N (M %)	N (M %)	N (M %)	M/N	M/N	M/N	M/N	M/N	M/N
Rathi 2002 [30]	USA	Caucasians	MSP	Tissue	49 (18.4)	-	16 (0)	-	-	-	-	-	-	-
Caceres 2004 [29]	USA	Caucasians	MSP	Tissue	35 (11.4)	10 (10)	10 (0)	10 (0)	0/12	4/23	1/8	3/27	2/21	2/14
Caceres 2004 [29]	USA	Caucasians	MSP	Blood	35 (8.6)	10 (10)	10 (0)	20 (0)	0/12	3/23	1/8	2/27	1/21	2/14
Makarla 2005 [28]	USA	Caucasians	MSP	Tissue	23 (21.7)	23 (0)	23 (0)	16 (0)	-	-	-	-	1/9	4/14
Tam 2007 [27]	China	Asians	MSP	Tissue	89 (47.2)	16 (50)	19 (26.3)	16 (25)	-	-	-	-	-	-
Wu 2007 [26]	Norway	Caucasians	MSP	Tissue	51 (23.5)	2 (0)	2 (0)	-	7/26	5/23	9/25	3/26	3/19	9/27
Shen 2007 [31]	China	Asians	MSP	Tissue	63 (22.2)	-	-	30 (0)	-	-	-	-	4/34	10/29
Sun 2008 [32]	China	Asians	MSP	Tissue	59 (32.2)	-	-	42 (2.4)	5/36	14/23	2/21	17/38	11/30	8/29
Ho 2010 [25]	China	Asians	MSP	Tissue	63 (23.8)	-	10 (0)	5 (0)	-	-	-	-	13/48	2/15
Bhagat 2012 [24]	India	Caucasians	MSP	Tissue	86 (29.1)	14 (28.6)	19 (15.8)	15 (0)	10/30	15/56	6/23	19/63	11/44	14/42
Zhang 2013 [23]	China	Asians	MSP	Blood	20 (35)	-	-	62 (0)	-	-	-	-	-	-
Brait 2013 [22]	USA	Caucasians	QMSP	Tissue	33 (15.1)	-	-	13 (0)	-	-	-	-	-	-
Al-Shabanah 2014 [21]	Saudi Arabia	Caucasians	MSP	Tissue	200 (36)	-	-	200 (10)	-	-	28/103	44/97	-	-

*MSP* methylation specific polymerase chain reaction, *QMSP* quantitative methylation specific PCR, *M* methylation, *N* the number of the total samples, “-” stands for data not applicable, *LMP* low malignant potential tumor

### The association of *APC* promoter hypermethylation and ovarian cancer

When cancer patients were compared to normal samples, benign lesions, and LMP patients, the fixed-effects model was used in the current meta-analysis, indicating a lack of heterogeneity (I^2^ = 0.0 %, *P* = 0.679; I^2^ = 0.0 %, *P* = 0.938; I^2^ = 0.0 %, *P* = 0.663; respectively) (Table 2).

**Table 2:** Summary of the pooled OR

	Studies	Overall OR (95 % CI)	I^2^; p	P value	Cases	Controls	p (Egger’s test)
Cancer vs. Normal	10	6.18 (4.02–9.51)	0.0 %; 0.679	<0.001	706	429	0.197
Subgroup							
Sample type							
Blood	2	18.71 (2.41–145.20)	39.6 %; 0.198	0.005	55	82	
Tissue	9	5.74 (3.68–8.95)	0.0 %; 0.818	< 0.001	651	347	
Race							
Caucasians	5	5.39 (3.25–8.94)	0.0 %; 0.981	< 0.001	294	155	
Asians	5	8.34 (3.63–19.13)	40.1 %; 0.154	< 0.001	567	274	
Cancer vs. Benign	7	3.26 (1.65–6.44)	0.0 %; 0.938	0.001	431	109	0.172
Cancer vs. LMP	5	1.30 (0.67–2.51)	0.0 %; 0.663	0.436	319	75	0.199
Clinicopathological features					Cancer patients		
					Low-grade	High-grade	
Tumor grade	4	0.46 (0.13–1.65)	68.5 %; 0.013	0.233	116	148	0.488
					StageI-II	Stage III-IV	
Tumor stage	5	0.76 (0.31–1.88)	61.0 %; 0.025	0.558	188	278	0.449
					Serous carcinoma	Non-serous carcinoma	
Tumor histology	7	0.56 (0.35–0.91)	0.0 %; 0.584	0.02	193	217	0.238

*LMP* low malignant potential tumor, *OR* odds ratio, *95 % CI* 95 % confidence interval

**Fig. 2:**
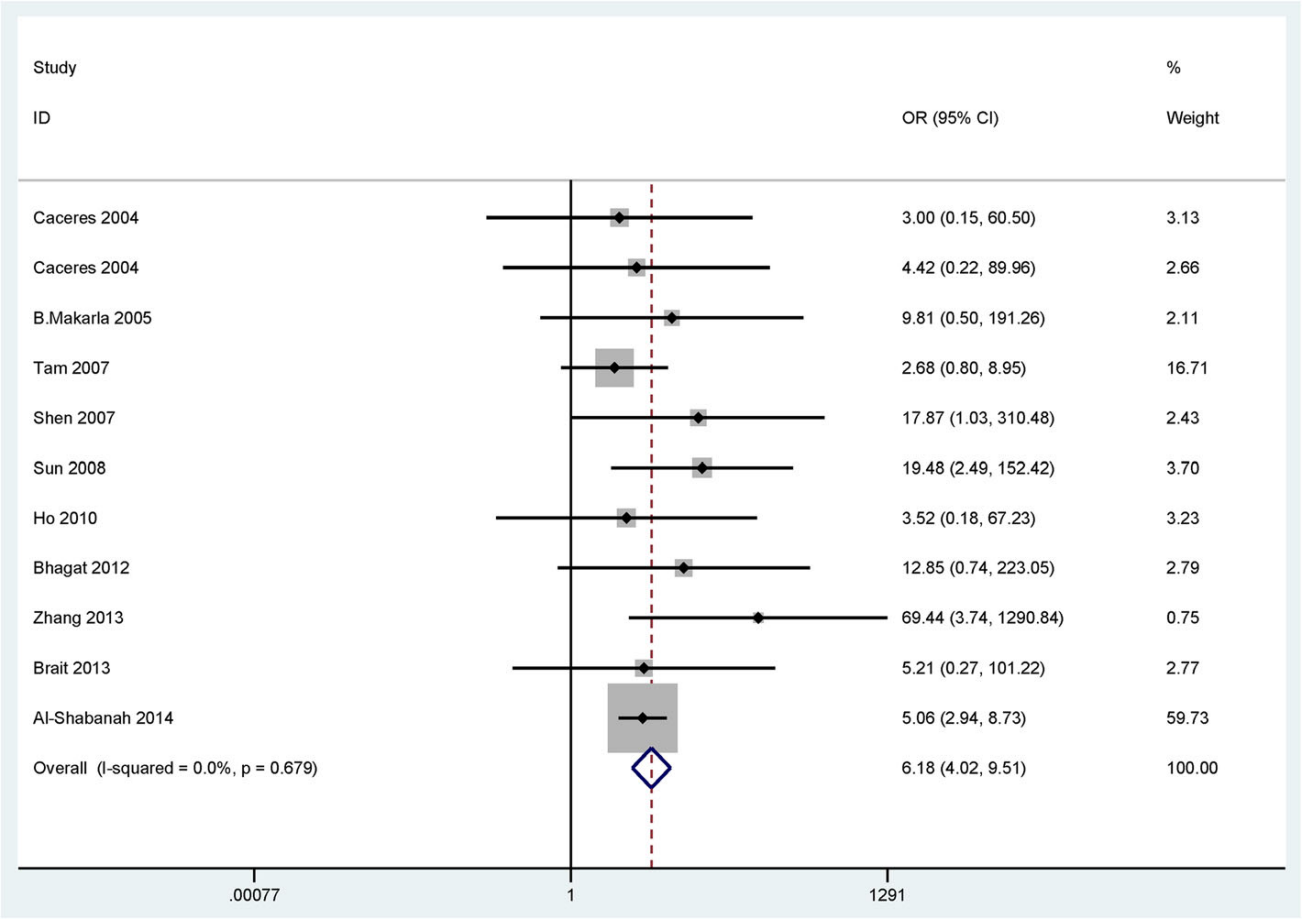
Forest plot for the association of *APC* promoter hypermethylation showing the pooled OR in cancer vs. normal controls

**Fig. 3:**
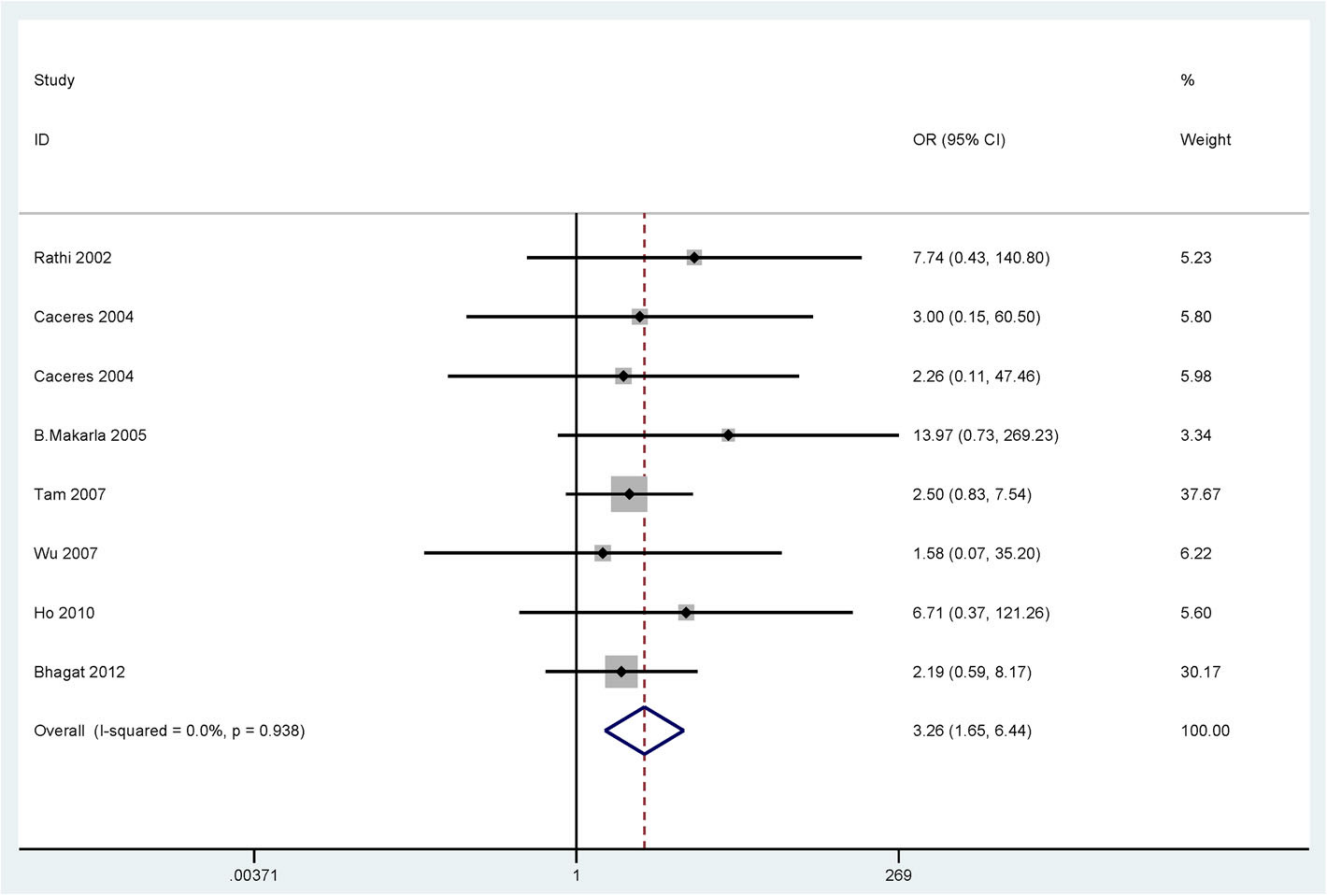
Forest plot for the association of *APC* promoter hypermethylation showing the pooled OR in cancer vs. benign controls

The results showed that the pooled OR of the *APC* promoter hypermethylation was significantly higher in ovarian cancer than in normal samples and benign lesions (OR = 6.18, 95 % CI = 4.02–9.51, *P* < 0.001; OR = 3.26, 95 % CI = 1.65–6.44, *P* = 0.001; respectively), including 10 studies of 706 ovarian cancer patients and 429 normal samples and 7 studies of 431 ovarian cancer patients and 109 benign lesions (Figs. 2 and 3).

In the comparison of cancer and normal controls, subgroup analyses based on race (Asians and Caucasians) and sample type (tissue and blood) were performed to assess the difference of a strong association. According to the subgroup analysis of the ethnic population, the result suggested that *APC* promoter hypermethylation was significantly associated with Asians and Caucasians (OR = 8.34, 95 % CI = 3.63–19.13, *P* < 0.001; OR = 5.39, 95 % CI = 3.25–8.94, *P* < 0.001; respectively). Subgroup analysis based on sample type showed that the pooled OR value was 18.71 (95 % CI, 2.41–145.20; *P* = 0.005) in blood and 5.74 (95 % CI, 3.68–8.95; *P* < 0.001) in tissue, indicating that the result was significantly associated with different sample types.

In addition, when 319 cancer patients were compared to 75 LMP patients from 5 studies, no significant association was observed in our study (OR = 1.30, 95 % CI = 0.67–2.51, *P* = 0.436) (Table 2).

### The association of *APC* promoter hypermethylation with clinicopathological features

We further analyzed the possible association between *APC* promoter hypermethylation and clinicopathological features. As shown in Table 2, there was statistically significant heterogeneity in *APC* promoter hypermethylation in ovarian cancer in relation to tumor grade and tumor stage (I^2^ = 68.5 %, *P* = 0.013; I^2^ = 61.0 %, *P* = 0.025; respectively), using the random-effects model. No obvious heterogeneity was found in relation to tumor histology, thus, a fixed-effects model was used (I^2^ = 0.0 % and *P* = 0.584).

**Fig. 4:**
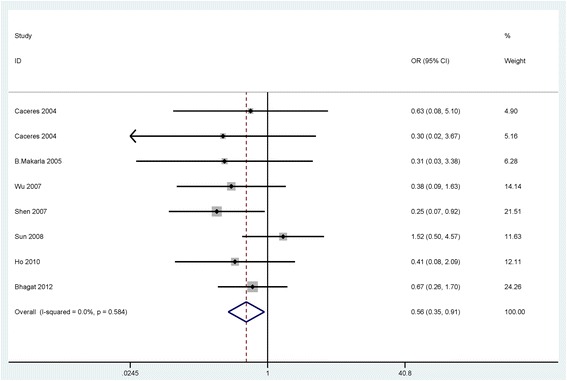
Forest plot for the correlation of *APC* promoter hypermethylation showing the pooled OR in cancer in relation to tumor histotype

The pooled OR from 4 studies involving 116 low-grade ovarian cancer patients and 148 high-grade ovarian cancer patients was shown in Table 2 (OR = 0.46, 95 % CI = 0.13–1.65, *P* = 0.233), suggesting that *APC* promoter hypermethylation was not significantly associated with tumor grade. As shown in Table 2, our result from 5 studies indicated that the correlation between *APC* promoter hypermethylation and tumor stage was not statistically significant (OR = 0.76, 95 % CI = 0.31–1.88, *P* = 0.558), including 188 early ovarian cancer patients and 278 advanced ovarian cancer patients. The overall OR from 7 studies including 193 serous carcinoma and 217 non-serous carcinoma cases suggested that *APC* promoter hypermethylation was significantly correlated with tumor histology (OR = 0.56, 95 % CI = 0.35–0.91, *P* = 0.02), and it was lower in serous carcinoma than in non-serous carcinoma (Fig. 4).

### Publication bias

Egger’s linear regression test was performed to detect the potential publication bias. The results of Egger’s test on *APC* promoter hypermethylation indicated that there was not obvious evidence of publication bias in the comparison of cancer and control groups, in relation to tumor grade, tumor stage, and tumor histology in cancer (all *P* > 0.05) (Table 2).

## Discussion

The loss of gene expression associated with the CpG islands of promoter methylation of different genes has been found in many cancers [33–35]. The promoter hypermethylation of tumor suppressor genes (TSG) involving cell proliferation, cell death, cell migration, and cell invasion leads to the initiation and development of cancer [36]. *APC* promoter hypermethylation has been reported in some cancers, including ovarian cancer, which indicates that it can become a noninvasive biomarker for cancer detection [23, 37–39]. Although some studies have been conducted to assess the frequency of hypermethylation of the *APC* promoter in OC, the results were still inconsistent and controversial. For example, Caceres et al. [29] found that the frequency of *APC* promoter hypermethylation was 11.4 % in ovarian cancer tissue. Tam et al. [27] found that frequency of *APC* promoter hypermethylation was 47.2 % in ovarian cancer tissue. Therefore, we utilized this meta-analysis to identify the correlation between *APC* promoter hypermethylation and OC.

Our findings supported that OC had a greater hypermethylation of *APC* promoter than benign lesions and normal samples, suggesting that *APC* inactivation via hypermethylation is involved in the carcinogenesis and development of OC. However, the frequency of *APC* promoter hypermethylation was similar in OC and LMP, indicating that *APC* promoter hypermethylation could not distinguish OC and LMP. Our results were credible based on the lack of publication bias.

When cancer was compared to normal controls, subgroup analysis of ethnicity showed that the pooled OR from the Asian and Caucasian populations was similar, suggesting that Asian and Caucasian populations were susceptible to *APC* promoter hypermethylation. Interestingly, subgroup analysis of sample type indicated that hypermethylated *APC* was significantly higher in blood (OR = 18.71, *P* = 0.005) than in tissue (OR = 5.74, *P* < 0.001), which suggested that *APC* promoter hypermethylation may become a potential noninvasive biomarker based blood test for OC. However, the results should be carefully considered as only two studies with a small number of subjects were included in the blood subgroup.

Next, the clinical significance of *APC* promoter hypermethylation was first determined in cancer. *APC* promoter hypermethylation was not correlated with clinical stage and tumor grade, but was associated with histological subtype in which it was significantly lower in serous carcinoma than in non-serous carcinoma, indicating that *APC* promoter hypermethylation was correlated with a decreased risk of serous carcinoma. Therefore, *APC* promoter hypermethylation may be a potential drug target for serous carcinoma.

The current study had several potential limitations. First, although we searched the literature as completely as possible, only articles published in English or Chinese were selected, which may lead to selection bias. Second, based on insufficient data and studies about other clinicopathological features, such as age, lymph node status, etc., the correlation between *APC* promoter hypermethylation and other clinicopathological features was not conducted in the present meta-analysis. Third, the interpretation of the pooled OR of the blood subgroup with a small sample size should be conservative. More studies comprising larger sample sizes are necessary to confirm our results.

## Conclusion

In conclusion, the current findings revealed that *APC* promoter hypermethylation may play a key role in the initiation of OC. *APC* promoter hypermethylation decreased the risk of the serous carcinoma histotype. Moreover, hypermethylated *APC* may be a potential promising biomarker for the clinical screening of OC in blood. Based on the limitations of the current sample size, additional studies is very essential in the future.

## Abbreviations

APC: The adenomatous polyposis coli
CC: Clear cell carcinoma
CI: Confidence interval
EC: Endometrioid carcinoma
LMP: Low malignant potential
MC: Mucinous carcinoma
OC: Ovarian cancer
OR: Odds ratio
PCR: Polymerase chain reaction
MSP: Methylation specific PCR
QMSP: Quantitative methylation specific polymerase chain reaction
TC: Transitional cell carcinoma
TSGs: Tumor suppressor genes

## References

[1] Siegel RL, Miller KD, Jemal A (2016). Cancer statistics, 2016. CA Cancer J Clin.

[2] Kurman RJ (2013). Origin and molecular pathogenesis of ovarian high-grade serous carcinoma. Ann Oncol.

[3] Kaja S, Hilgenberg JD, Collins JL, Shah AA, Wawro D, Zimmerman S (2012). Detection of novel biomarkers for ovarian cancer with an optical nanotechnology detection system enabling label-free diagnostics. J Biomed Opt.

[4] Lehtinen L, Vesterkvist P, Roering P, Korpela T, Hattara L, Kaipio K (2016). Reg4 is highly expressed in mucinous ovarian cancer: A potential novel serum biomarker. PLoS One.

[5] Khan SA, Reddy D, Gupta S (2015). Global histone post-translational modifications and cancer: Biomarkers for diagnosis, prognosis and treatment?. World J Biol Chem.

[6] Ngollo M, Dagdemir A, Karsli-Ceppioglu S, Judes G, Pajon A, Penault-Llorca F (2014). Epigenetic modifications in prostate cancer. Epigenomics.

[7] Paska AV, Hudler P (2015). Aberrant methylation patterns in cancer: A clinical view. Biochem Med (Zagreb).

[8] Ghavifekr Fakhr M, Farshdousti Hagh M, Shanehbandi D, Baradaran B (2013). DNA methylation pattern as important epigenetic criterion in cancer. Genet Res Int.

[9] Sowa Y, Sakai T (2015). Development of novel epigenetic molecular-targeting agents. Nihon Rinsho.

[10] Ouadid-Ahidouch H, Rodat-Despoix L, Matifat F, Morin G, Ahidouch A (1848). DNA methylation of channel-related genes in cancers. Biochim Biophys Acta.

[11] Bustaffa E, Stoccoro A, Bianchi F, Migliore L (2014). Genotoxic and epigenetic mechanisms in arsenic carcinogenicity. Arch Toxicol.

[12] Friedrich A, Kullmann F (2003). Familial adenomatous polyposis syndrome (fap): Pathogenesis and molecular mechanisms. Med Klin (Munich).

[13] Nelson S, Nathke IS (2013). Interactions and functions of the adenomatous polyposis coli (apc) protein at a glance. J Cell Sci.

[14] Aceto GM, Fantini F, De Iure S, Di Nicola M, Palka G, Valanzano R (2015). Correlation between mutations and mrna expression of apc and mutyh genes: New insight into hereditary colorectal polyposis predisposition. J Exp Clin Cancer Res.

[15] Kamory E, Olasz J, Csuka O (2008). Somatic apc inactivation mechanisms in sporadic colorectal cancer cases in hungary. Pathol Oncol Res.

[16] Fodde R, Kuipers J, Rosenberg C, Smits R, Kielman M, Gaspar C (2001). Mutations in the apc tumour suppressor gene cause chromosomal instability. Nat Cell Biol.

[17] Coory MD (2010). Comment on. Heterogeneity in meta-analysis should be expected and appropriately quantified. Int J Epidemiol.

[18] Higgins JP, Thompson SG, Deeks JJ, Altman DG (2003). Measuring inconsistency in meta-analyses. BMJ.

[19] DerSimonian R (1996). Meta-analysis in the design and monitoring of clinical trials. Stat Med.

[20] Peters JL, Sutton AJ, Jones DR, Abrams KR, Rushton L (2006). Comparison of two methods to detect publication bias in meta-analysis. JAMA.

[21] Al-Shabanah OA, Hafez MM, Hassan ZK, Sayed-Ahmed MM, Abozeed WN, Alsheikh A (2014). Methylation of sfrps and apc genes in ovarian cancer infected with high risk human papillomavirus. Asian Pac J Cancer Prev.

[22] Brait M, Maldonado L, Noordhuis MG, Begum S, Loyo M, Poeta ML (2013). Association of promoter methylation of vgf and pgp9.5 with ovarian cancer progression. PLoS One.

[23] Zhang Q, Hu G, Yang Q, Dong R, Xie X, Ma D (2013). A multiplex methylation-specific pcr assay for the detection of early-stage ovarian cancer using cell-free serum DNA. Gynecol Oncol.

[24] Bhagat R, Chadaga S, Premalata CS, Ramesh G, Ramesh C, Pallavi VR (2012). Aberrant promoter methylation of the rassf1a and apc genes in epithelial ovarian carcinoma development. Cell Oncol (Dordr).

[25] Ho CM, Lai HC, Huang SH, Chien TY, Lin MC, Chang SF (2010). Promoter methylation of sfrp5 in patients with ovarian clear cell adenocarcinoma. Eur J Clin Invest.

[26] Wu Q, Lothe RA, Ahlquist T, Silins I, Trope CG, Micci F (2007). DNA methylation profiling of ovarian carcinomas and their in vitro models identifies hoxa9, hoxb5, scgb3a1, and crabp1 as novel targets. Mol Cancer.

[27] Tam KF, Liu VW, Liu SS, Tsang PC, Cheung AN, Yip AM (2007). Methylation profile in benign, borderline and malignant ovarian tumors. J Cancer Res Clin Oncol.

[28] Makarla PB, Saboorian MH, Ashfaq R, Toyooka KO, Toyooka S, Minna JD (2005). Promoter hypermethylation profile of ovarian epithelial neoplasms. Clin Cancer Res.

[29] Ibanez de Caceres I, Battagli C, Esteller M, Herman JG, Dulaimi E, Edelson MI (2004). Tumor cell-specific brca1 and rassf1a hypermethylation in serum, plasma, and peritoneal fluid from ovarian cancer patients. Cancer Res.

[30] Rathi A, Virmani AK, Schorge JO, Elias KJ, Maruyama R, Minna JD (2002). Methylation profiles of sporadic ovarian tumors and nonmalignant ovaries from high-risk women. Clin Cancer Res.

[31] Sheng WJ, Guo KJ, Song S, Dai DQ (2007). Clinical value of promoter hypermelhylaIion of APC gene in epithelial ovarian carcinoma. Shandong Med.

[32] Sun J, Lu J, Guo DF, Wu GP (2008). Hypermethylation of APC gene promoter in epithelial ovarian carcinoma. J Bethune Military Med Coll.

[33] Huang T, Chen X, Hong Q, Deng Z, Ma H, Xin Y (2015). Meta-analyses of gene methylation and smoking behavior in non-small cell lung cancer patients. Sci Rep.

[34] Ng JM, Yu J (2015). Promoter hypermethylation of tumour suppressor genes as potential biomarkers in colorectal cancer. Int J Mol Sci.

[35] Herman JG, Baylin SB (2000). Promoter-region hypermethylation and gene silencing in human cancer. Curr Top Microbiol Immunol.

[36] Maziveyi M, Alahari SK (2015). Breast cancer tumor suppressors: A special emphasis on novel protein nischarin. Cancer Res.

[37] Huang KT, Mikeska T, Li J, Takano EA, Millar EK, Graham PH (2015). Assessment of DNA methylation profiling and copy number variation as indications of clonal relationship in ipsilateral and contralateral breast cancers to distinguish recurrent breast cancer from a second primary tumour. BMC Cancer.

[38] Balgkouranidou I, Matthaios D, Karayiannakis A, Bolanaki H, Michailidis P, Xenidis N (2015). Prognostic role of apc and rassf1a promoter methylation status in cell free circulating DNA of operable gastric cancer patients. Mutat Res.

[39] Dumitrescu RG (2012). Epigenetic markers of early tumor development. Methods Mol Biol.

